# Abundance of Amino Acid Transporters and mTOR Pathway Components in the Gastrointestinal Tract of Lactating Holstein Cows

**DOI:** 10.3390/ani13071189

**Published:** 2023-03-29

**Authors:** Qianming Jiang, Danielle N. Sherlock, Jessie Guyader, Juan J. Loor

**Affiliations:** 1 Department of Animal Sciences, University of Illinois, Urbana, IL 61801, USA; 2 Evonik Operations GmbH, Hanau-Wolfgang, 63457 Essen, Germany; 3 Division of Nutritional Sciences, University of Illinois, Urbana, IL 61801, USA

**Keywords:** amino acid concentration, rumen, duodenum, jejunum, ileum, lactation

## Abstract

**Simple Summary:**

Peptides and amino acids (AAs) arising from feed or microbial protein digestion require transporters in the gastrointestinal tract (GIT) for uptake into the blood. The mTOR pathway, considered the master regulator of protein synthesis, is partly controlled by specific AAs. We measured AA concentrations, mRNA abundance of AA transporters, and genes in the mTOR pathway in epithelia from the rumen, duodenum, jejunum, and ileum of lactating Holstein cows. The concentrations of most AAs and the abundances of AA transporters and mTOR mRNA were greater in the small intestine than in the rumen. As in non-ruminants, the absorption of AAs from the small intestine is partly due to the greater abundance of transporters. Compared with the ruminal epithelium, the greater abundance of mTOR in the small intestine underscored its role in regulating cellular protein synthesis.

**Abstract:**

Data from non-ruminants indicate that amino acid (AA) transport into cells can regulate mTOR pathway activity and protein synthesis. Whether mTOR is expressed in the ruminant gastrointestinal tract (GIT) and how it may be related to AA transporters and the AA concentrations in the tissue is unknown. Ruminal papillae and the epithelia of the duodenum, jejunum, and ileum collected at slaughter from eight clinically healthy Holstein in mid-lactation were used. Metabolites and RNA were extracted from tissue for liquid chromatography–mass spectrometry and RT-qPCR analysis. The glycine and asparagine concentrations in the rumen were greater than those in the intestine (*p* < 0.05), but the concentrations of other AAs were greater in the small intestine than those in the rumen. Among the 20 AAs identified, the concentrations of glutamate, alanine, and glycine were the greatest. The mRNA abundances of *AKT1* and *MTOR* were greater in the small intestine than those in the rumen (*p* < 0.05). Similarly, the *SLC1A1*, *SLC6A6*, *SLC7A8*, *SLC38A1*, *SLC38A7*, and *SLC43A2* mRNA abundances were greater (*p* < 0.05) in the small intestine than those in the rumen. The mRNA abundances of *SLC1A5*, *SLC3A2,* and *SLC7A5* were greater in the rumen than those in the small intestine (*p* < 0.05). Overall, the present study provides fundamental data on the relationship between mTOR pathway components and the transport of AAs in different sections of the gastrointestinal tract.

## 1. Introduction

In ruminants, the majority of microbial proteins and rumen-undegradable proteins are digested into amino acids (AAs) or peptides by enzymes in the abomasum, followed by their transport into the various sections of the small intestine. Thus, although most of the AA absorption from digesta occurs primarily in the ileum, the extent to which the ruminal and intestinal epithelia are equipped with AA transporters is still unknown. There are several types of AA transporters. For example, some transporters, such as *SLC1A1* and SLC1A5, are sodium-dependent [1,2] and the concentration gradient between cell membranes can drive absorption via these transporters. Some examples of sodium-independent AA transporters are *SLC7A5* (CD98LC, 4F2 light chain, LAT1, MPE16) and *SLC3A2* (4F2 cell-surface antigen heavy chain, 4F2hc, MDU1), both of which function as high-affinity transporters that mediate the uptake of large, neutral AAs, such as tryptophan (Trp), phenylalanine (Phe), leucine (Leu), and histidine (His) [3]. Although the mRNA abundance of selected AA transporters has been measured in adipose [4,5], placenta [6], mammary gland [7], and fetal intestinal tissue [8], a comprehensive examination across the different sections of the ruminant gut is not available.

Once AAs reach the ruminal cell or the enterocytes lining the small intestine, a portion can be used for cellular protein synthesis [9]. During protein synthesis, gene transcription is regulated by transcription factors, followed by translation, including three steps: initiation, elongation, and termination. In the initiation phase, the poly-A tails of mRNA bind to the poly(A)-binding protein (PABP), and the GTP cap binds to eukaryotic translation initiation factor 4E (eIF4E). Eukaryotic translation initiation factor 4 G (eIF4G) interacts with PABP and, along with eIF4E, forms an mRNA loop for translation. During the elongation step, eukaryotic translation elongation factor 2 (eEF2) controls the movement speed of the ribosome to construct the AAs into a chain. The stop codon terminates translation.

The mechanistic target of rapamycin kinase (mTOR; gene symbol *MTOR*), a serine/threonine–protein kinase, phosphorylates eIF4EBP and RPS6KB [10]. To inhibit the formation of the mRNA loop during the initiation of translation, eIF4EBP competes with eIF4G to bind eIF4E [11]. The phosphorylation of eIF4EBP prevents it from binding to the eIF4E; thus, mTOR enables the initiation of translation [12,13]. The phosphorylation of ribosomal protein S6 kinase (RPS6K) leads to the subsequent phosphorylation of eIF4E and ribosomal protein S6 (RPS6) [14]. The phosphorylated RPS6 increases the translation of mRNA [15], underscoring that RPS6K promotes transcriptional initiation and elongation. 

In order to have a blueprint of AA transport and utilization in the GIT of dairy cows, ruminal papillae and epithelia of the duodenum, jejunum, and ileum collected at slaughter from Holstein cows in mid-lactation were used. Metabolites and RNA were extracted from the tissue for liquid chromatography–mass spectrometry and RT-qPCR analysis. The central objective was to assess the relationship between the mTOR pathway components and transport of AA in different sections of the gastrointestinal tract.

## 2. Materials and Methods

### 2.1. Animal Handling and Experimental Design

The Institutional Animal Care and Use Committee (IACUC) at the University of Illinois approved the procedures (#19161). Eight clinically healthy Holstein mid-lactating cows housed with other cows in the University of Illinois Dairy Unit herd in free stalls were selected. All cows were milked twice per day. Their diet contained 17.4% crude protein and 1.74 Mcal/kg net energy for lactation. The cows were fed at 06:00 and 17:30 h daily. On the day of sacrifice at 06:00 h, each cow received 300 mg of xylazine via intramuscular injection for sedation (Rompun^®^ 100 mg/m, Dechra, Kansas City, KS, USA). The cows were then placed in a livestock trailer (EBY Maverick LS livestock trailer, EBY, Seymour, IN, USA) and transported 0.8 km from the University of Illinois Dairy Unit to the Veterinary Diagnostics Laboratory at the University of Illinois College of Veterinary Medicine in Urbana-Champaign. The cows became recumbent within 10 min of injection and were then euthanized with a penetrating captive bolt. The cows were removed from the trailer and then exsanguinated, and within 10 min, the body cavity was opened.

### 2.2. Sample Collection

Within 20 min of the animal’s death, tissue samples from the rumen, duodenum, jejunum, and ileum were collected. Ruminal papillae were harvested from the rumen’s ventral sac using surgical scissors. The small intestine was cut from the rumen and placed on a necropsy table, where duodenal tissue was collected approximately 25 cm distal from the pyloric sphincter; jejunum was collected approximately 1 m proximal to the ileocecal junction, and the ileum was collected approximately 18 cm proximal to the ileocecal junction [16]. Twenty-five-centimeter segments from the duodenum, jejunum, and ileum were cut into pieces measuring approximately 10 cm × 20 cm and washed with phosphate-buffered saline. Then, a sterile scalpel blade was used to scrape the epithelium. Cryovials were used to collect samples, which were then quickly frozen in liquid nitrogen. After transporting them to the lab, the tissues were stored at −80 °C until use.

### 2.3. Metabolomics

With 4 mL/g of cold methanol and 0.85 mL/g of cold water, approximately 100 mg of the frozen sample was homogenized. The homogenate was vortexed with 4 mL/g chloroform and 2 mL/g for 60 s, left on ice for 10 min to partition, and centrifuged for 10 min at 4 °C at 12,000× *g*. The supernatant was collected and separated into 2 aliquots. The first part was used to determine the protein concentration via the Bradford assay (no. 500–0205, Bio-Rad Laboratories Inc., Hercules, CA, USA). The second aliquot was delivered to the Metabolomics Unit at the Roy J. Carver Biotechnology Center (University of Illinois, Urbana) for analysis. Targeted metabolomics (liquid chromatography (LC–MS)) was performed to quantify specific AAs via a targeted amino acid assay [17]. A commercial amino acid standard solution (AAS18, Sigma, St. Louis, MO, USA) was used to generate calibration curves. Twenty microliters of internal standard (DL-Chlorophenyl alanine, 0.01 mg/mL) were spiked into the samples at the beginning of extraction. Chromatography was performed on a Vanquish LC system (Thermo Scientific, Waltham, MA, USA) and a TSQ Altis LC-MS mass spectrometer system (Thermo Scientific). Data were acquired in both positive and negative (Taurine) SRM modes. Peak integration and quantitation using calibration curves adjusted for internal standards were performed using the Thermo TraceFinder (4.1) software. Cysteine was not detectable with this assay. However, Cys was identified via targeted LC-MS metabolite profiling with the Agilent 1200 HPLC system (Agilent Technologies, Santa Clara, CA, USA) on a Phenomenex Luna C18 column (4.6 × 150 mm, 5 μm; Phenomenex, Torrance, CA, USA) and a 5500 QTRAP system mass spectrometer (Sciex, Framingham, MA, USA). Data were acquired under positive and negative electrospray ionization. Analyst 1.7.1 software (Agilent) was used for data acquisition and analysis. More details are reported in the Supplementary Materials File.

### 2.4. RNA Extraction

Approximately 50 mg of tissue was homogenized with 1 mL of Qiazol (Qiagen, Hilden, Germany). After centrifuging the samples for 10 min at 12,000× *g* and 4 °C, the supernatant was collected and kept at room temperature for 5 min before the addition of 200 µL chloroform. The samples were then shaken manually and kept at room temperature for 3 min before centrifugation for 15 min at 12,000× *g* at 4 °C. The collected supernatant was mixed with 750 µL of ethanol. These reagents and materials were from the RNAase kit (Qiagen, Hilden, Germany). Details of the RNAase kit’s operation are in the Supplementary Materials File. Total RNA quantification was conducted using a Nanodrop ND-1000 (NanoDrop Technologies, Rockland, DE). The RNA was diluted to 100 ng/µL with DNase/RNase-free water. The purity and integrity of the extracted RNA were evaluated using an Agilent Bioanalyzer in the Roy J. Carver Biotechnology Center, University of Illinois, Urbana-Champaign. Ruminal, duodenal, jejunal, and ileal samples had average RNA quality numbers (RQNs) of 8.1 ± 0.7, 7.4 ± 0.9, 7.0 ± 0.7, and 7.1 ± 0.5, respectively. The average 28 s/18 s values were 1.5 ± 0.3, 1.1 ± 0.3, 1.1 ± 0.2, and 1.0 ± 0.2, respectively.

### 2.5. cDNA Synthesis and qRT-PCR

Eight microliters of 100 ng RNA/µL plus 80 µL of 0.00035 mg/µL Random Primers (11034731001, Roche, Basel, Switzerland) were incubated at 65 °C for 5 min. Seventy-two microliters of master mix composed of 4 μL of 5X First-Strand Buffer (EP0442, Thermo Scientific), 1 μL of Oligo dT18 (Integrated DNA Technologies), 2 µL of 10 mM dNTP mix (18427088, Invitrogen, Waltham, MA, USA), 0.25 μL (50 U) of Revert aid (EP0442, Thermo Scientific), 0.125 μL of RiboLock RNase Inhibitor (EO0381, Thermo Scientific), and 1.625 µL of DNase/RNase-free water were added to each sample and the reaction was performed with the following temperature program: 25 °C for 5 min, 42 °C for 60 min, and 70 °C for 5 min. The cDNA was then diluted 1:3 with DNase/RNase-free water. Quantitative PCR (qPCR) was performed as reported in our previous papers with *GAPDH*, *UXT*, and *RPS9* (commonly used in bovine gene work) as the internal control genes [18,19,20] using the QuantStudio Software (version v1.7.2, Applied Biosystems, Foster City, CA, USA). For reference, *UXT* and *RPS9* were deemed suitable internal control genes in the ruminal epithelium of cows and in various tissues from buffalo [21]. *GAPDH* was determined to be a stable internal control gene and has been used in several studies of the GIT in cattle [22,23,24,25]. The quantity data for the target genes obtained from QuantStudio Software were normalized by dividing the geometric mean of the three internal control genes. Specific details are reported in the Supplementary Materials File. The primers for the target genes were from one of our previous papers [6].

### 2.6. Statistical Analysis

All data were checked for normality via the Shapiro–Wilk test and analyzed with the MIXED procedure in SAS 9.4 (SAS Institute Inc., Cary, NC, USA). The model included the fixed effect of GIT tissue section and the random effect of cow. The preplanned CONTRAST *P* values for comparing the rumen versus small intestine (duodenum, jejunum, ileum), the duodenum versus jejunum and ileum, and the jejunum versus ileum were used to determine statistical significance. Tukey’s multiple comparison test was used to determine differences across the different sections of the GIT. MATLAB was used to read the SAS results to extract the least squares means, standard error of the mean, and *p*-value. The least-square means (LSMs) of the AA concentrations and the relative mRNA abundances were imported into Genesis v. 1.8.1 [26] for hierarchical clustering using the average linkage weighted pair group method with arithmetic mean (WPGMA).

## 3. Results

### 3.1. Amino Acid Profiles

**Figure 1:**
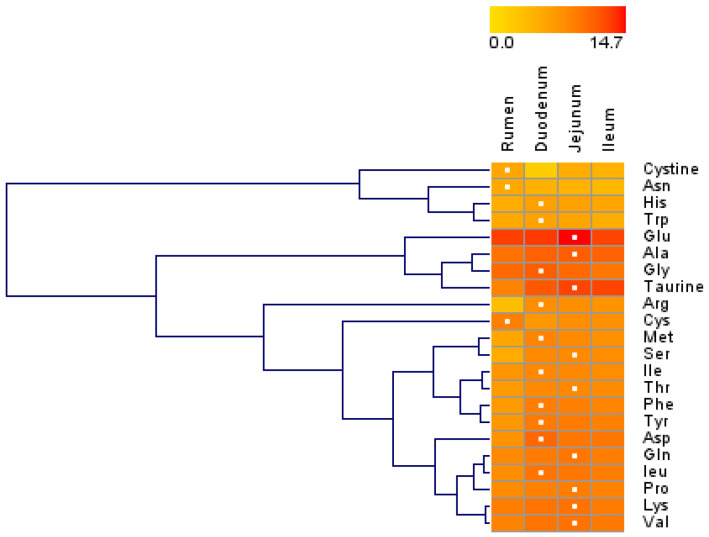
Hierarchical clustering of amino acid concentrations in tissue harvested from the rumen, duodenum, jejunum, and ileum of lactating Holstein cows (*n* = 8/group). Dendrograms allow the visualization of clusters of similarity in concentration patterns (links denoted by the lines at the left side of the picture). Concentration levels are denoted by shades of yellow and red according to the log2-transformed least-squares mean. Red intensity denotes a high concentration and yellow intensity a low concentration. White dots denote the highest concentration of an amino acid in a given tissue.

Overall, most AA concentrations were greater in the small intestine than in the rumen, and the three sections of the small intestine had similar AA concentrations (Table 1). Except for cystine and glutamate (Glu), the concentrations of all other AAs differed among the rumen, duodenum, jejunum, and ileum (*p* < 0.05). Except for Trp, the concentrations of other AAs differed between the rumen and small intestine (*p* < 0.05). The asparagine (Asn) and cysteine (Cys) concentrations in the rumen were greater than those in the small intestine (*p* < 0.05), while the concentrations of other AAs in the rumen were lower than those in the small intestine (*p* < 0.05). The aspartate (Asp), glycine (Gly), Met, Phe, taurine, and Trp concentrations in the duodenum were greater (*p* < 0.05) than those in the combination of the jejunum and ileum, while the taurine concentrations in the combination of the jejunum and ileum were ~3 to 4-fold greater (*p* < 0.05) than that in the duodenum. The hierarchical clustering of the AA concentrations revealed similar patterns of AA concentrations (Figure 1). The concentrations of cystine, Asn, His, and Trp were in a large cluster with similar patterns, and their concentrations were lower than 1100 ng/mg of total protein. The concentrations of Glu, Ala, Gly, and taurine (second main cluster) were relatively high among all AAs, and their concentrations in the rumen were lower than those in the small intestine.

### 3.2. mRNA Abundance of Protein Synthesis Regulation Genes

The *MTOR* mRNA abundance in the rumen was lower than that in the small intestine (*p* < 0.05), and its abundance in the jejunum and ileum was greater than that in the duodenum (*p* < 0.05). The *AKT1* mRNA abundance was greater in the small intestine than that in the rumen (*p* < 0.05). The *EEF1A1* mRNA abundance in the rumen was greater than that in the small intestine (*p* < 0.05). The differences in mRNA abundance of *RPS6KB1* and *IRS1* were not significant among these four sections of the GIT.

### 3.3. mRNA Abundance of Amino Acid Transporters

**Figure 2:**
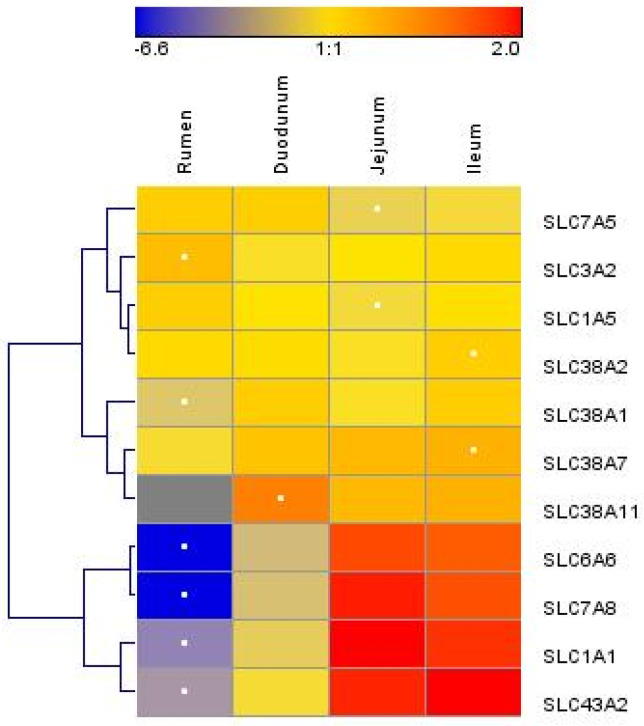
Hierarchical clustering of amino acid transporter abundance data in tissue harvested from the rumen, duodenum, jejunum, and ileum of lactating Holstein cows (*n* = 8/group). Dendrograms allow the visualization of clusters of similarity in abundance patterns among genes between treatments (links denoted by the lines at the left side of the picture). Cutoffs for abundance level are denoted by the shades of blue, yellow, and red according to the log2-transformed least-square mean. Red intensity denotes high expression, and blue intensity indicates low expression. White dots denote the lowest or highest mRNA abundance for each gene.

We measured the mRNA abundances of 11 AA transporters. The mRNA abundances of solute carrier family 1 member 1 (*SLC1A1*), *SLC3A2*, solute carrier family 6 member 6 (*SLC6A6*), *SLC7A5*, solute carrier family 7 member 8 (*SLC7A8*), solute carrier family 38 member 1 (*SLC38A1*), solute carrier family 38 member 7 (*SLC38A7*), and solute carrier family 43 member 2 (*SLC43A2*) were significantly different between the rumen, duodenum, jejunum, and ileum (*p* < 0.05) (Table 2). The mRNA abundances of *SLC1A1*, *SLC6A6*, *SLC7A8*, *SLC38A1*, *SLC38A7*, and *SLC43A2* were greater (*p* < 0.05) in the small intestine than those in the rumen, while the *SLC1A5*, *SLC3A2,* and *SLC7A5* mRNA abundances were greater in the rumen than those in the small intestine (*p* < 0.05). The *SLC1A1*, *SLC3A2*, *SLC6A6*, *SLC7A5*, *SLC7A8*, and *SLC43A2* abundances were significantly different between the duodenum and the combination of the jejunum and ileum (*p* < 0.05). There were two main clusters of AA transporters (Figure 2). The first cluster included *SLC7A5*, *SLC3A2*, *SLC1A5*, *SLC38A2*, *SLC38A1*, *SLC38A7*, and *SLC38A11*, and the second cluster included *SLC6A6*, *SLC7A8*, *SLC1A1*, and *SLC43A2*. Overall, the AA transporter mRNA abundance was low in the rumen and high in the jejunum and ileum, and in the second cluster, the variation in mRNA abundance was larger among different sections of the GIT.

## 4. Discussion

### 4.1. Amino Acid Profiles

After AAs are transported from the gut or the blood [27] into gut tissue, they can be metabolized as an energy source or used for other processes, such as protein synthesis [28,29]. Studies of AA metabolism in the rumen have mainly focused on the role of the microbiome [30], with few data available on AA metabolism within ruminal tissue. Most data on AA metabolism in GIT tissue are from non-ruminant studies. For instance, asparaginases have been detected in the small intestines of dogs and guinea pigs, but not rats, indicating potential differences in AA metabolism across species [31]. Thus, the greater Asn concentrations in the rumen than those in the small intestine in the present study suggest that Asn metabolism might be different across the GIT. Tyrosine (Tyr) can be synthesized from Phe in the intestines of pigs, rats, and cattle [31], underscoring the similarity in the metabolism of this AA across species. Recent data using [^13^C]Phe injected into the external jugular vein underscored that the ruminal epithelium relies on AAs for protein synthesis [32]. The catabolism of Met by the GIT, at least in nonrumiants, is highlighted by data demonstrating that parenterally fed (intravenous administration) pigs exhibited a 30% reduction in Met requirements than enterally fed pigs, regardless of dietary Cys supply [33,34]. A subsequent study with multicatheterized pigs confirmed that approximately 30% of dietary Met is metabolized by the intestine [27,35]. Our previous study indicated that a fraction of Met is potentially used to synthesize Cys in the GIT through reactions in one-carbon metabolism [19]. The ruminal epithelium is composed of highly keratinized, stratified squamous cells [36] that are rich in Cys (at least in humans) [37]; thus, the presence of keratin in the ruminal epithelium might be the reason for the markedly high Cys concentrations in this tissue. 

In non-ruminant animals, the intestinal mucosa metabolizes over 95% of the enteral Glu [38]. In fact, both Glu and glutamine (Gln) are metabolized by intestinal enterocytes for protein synthesis or as a source of ATP [39]. In the jejunum of rats, 66, 98, and >99% of luminal Gln, Glu, and aspartate, respectively, were catabolized [9]. Besides ATP, the metabolism of Glu by the intestine leads to the production of Pro, Arg, Ala, citrulline, and glutathione [38]. In isolated ruminal epithelial and duodenal mucosal cells from beef cattle, Glu contributed to a large amount of alpha-ketoglutarate in the tricarboxylic acid (TCA) cycle for ATP production [40]. Thus, relative to other AAs that were detected, the high concentration of Glu in the rumen and intestine seems to agree with these previous findings. Similarly, the fact that Gln only contributed a small fraction of the alpha-ketoglutarate [40] could explain the low concentration of Gln in our study. 

Approximately 44% of the branched-chain amino acids (BCAAs) were extracted by first-pass splanchnic metabolism in neonatal piglets [39]. In dogs, approximately 30% of Leu was extracted by the splanchnic tissue, of which ~45–55% was used for the synthesis of proteins and transamination to feed the TCA cycle [41]. In piglets, 40% of Leu was extracted by portal-drained viscera, and <20% of the extracted BCAAs was used for protein synthesis [42]. Clearly, the metabolism of BCAAs in ruminants versus non-ruminants differs [9] because leucine (Leu) catabolism is low relative to its use for protein synthesis in sheep [43]. It is possible that the lower concentrations of Leu, Ile, and valine (Val) in the rumen than those in the small intestine in the present study were associated with differences in the capacity of these tissues to metabolize BCAAs.

Forty percent and 38% of the luminal Arg in rats and humans was catabolized in the intestine, respectively, and the discovery of type II arginase and proline oxidase underscored the ability of intestinal mucosal in non-ruminants to metabolize Arg and Pro [9]. Furthermore, the expression of carbamoyl–phosphate synthetase I and ornithine carbamoyl transferase in rats indicated that the urea cycle is an active pathway in the intestinal mucosa [44]. Feeding Arg to feed-restricted ewes increased the Arg, ornithine, and citrulline concentrations in the fetal duodenum, jejunum, and ileum [8], confirming the urea cycle’s activity in the small intestine. Thus, we speculate that the higher concentrations of Arg and Asp in the small intestine than those in the rumen may be related to differences in urea cycle activity.

Although taurine is not usually a quantitatively important nutrient in the diet of ruminants [45], its concentration was very high in the intestine of dairy cows. One of our previous studies revealed the existence of taurine synthesis intermediates (cysteinesulfinic acid and hypotaurine) in the GIT of dairy cows [46,47] and the presence of cysteinesulfinic acid decarboxylase (CSAD), an enzyme in the taurine synthesis pathway [19]. These data suggested that taurine may be synthesized endogenously in the GIT. Taurine and glycine are conjugated to bile acids synthesized in the liver prior to their storage in the gall bladder and eventual secretion into the duodenum, where the gut microbiota deconjugates the bile salts into bile acids and AAs [48]. We detected higher taurocholic acid, glycocholic acid, and glycochenodeoxycholic acid concentrations in the duodenum than those in the rumen, jejunum, and ileum (Supplementary Figure S1). Although we are unaware of data on microbiota’s content of taurine, its concentration was the lowest in the rumen, while the jejunum and ileum had higher concentrations than those in the duodenum (Table 1). Thus, similar to humans, we hypothesize that the taurocholic acid secreted into the duodenum is deconjugated by the gut microbiota and the resulting taurine is likely absorbed mainly at the jejunum and ileum in dairy cows.

Although the creatine concentrations in the rumen and intestine were very high in a previous study from our laboratory [19], it is not a metabolite typically produced by plants [49], but is instead synthesized from Gly and Arg [50]. Thus, the high Arg and Gly concentrations in the duodenum and jejunum suggested that the intestine could synthesize creatine. This is further supported by the guanidinoacetate methyltransferase (GAMT) mRNA abundance in our previous study. As approximately 40 and 50% of dietary Ser and Gly are extracted in the first pass by portal-drained viscera in non-ruminants [9,42,51], it is possible that Gly and Arg in the present study were utilized by GAMT to synthesize creatine [50]. Physiologically, the synthesis of creatine in the gut would help to generate ATP and maintain intestinal homeostasis [52]. This idea is supported by the fact that close to 23% of the whole-body energy is consumed by the non-ruminant GIT [53]. Clearly, the ruminant GIT might also rely on creatine synthesis from AAs as a source of cellular ATP. 

### 4.2. mRNA Abundance of Targets Associated with Protein Synthesis

The mRNA abundances of α serine–threonine kinase 1 (AKT), ribosomal protein S6 kinase B1 (RPS6KB1), insulin receptor substrate 1 (IRS1), and eukaryotic translation initiation factor 4E-binding protein 1 (EIF4EBP1) were up-regulated by the supplementation of rumen-protected methionine (RPM) [4,5]. Similarly, the protein abundances of p-mTOR and RPS6 were greater with RPM supplementation, and the protein abundances of insulin receptor (INSR), AKT, p-AKT, p-mTOR, ribosomal protein S6 (RPS6), p-RPS6, phosphorylated eukaryotic translation elongation factor 2 (p-EEF2), and p-EIF4EBP1 changed significantly during the transition into lactation [4]. At the initiation of translation, the translation initiation protein complex, including eukaryotic translation initiation factor 4E (eIF4E), binds to mRNA to allow for the ribosome to translate. The eukaryotic translation initiation factor 4E-binding protein (EIF4EBP) binds to the EIF4E in the translation initiation protein complex, preventing translation initiation [54]. Thus, the lack of a difference in the *EIF4EBP1* and *EIF4EBP2* mRNA abundances across tissues suggested that the proteins encoded by these genes may work in a similar fashion to affect protein synthesis.

The kinase mTOR, which phosphorylates EIF4EBP1, prevents EIF4EBP1 binding to EIF4E and phosphorylates RPS6KB1, thus promoting protein synthesis [55]. AKT1 phosphorylates and activates mTOR. Insulin receptor substrate 1 (IRS1) is upstream of the AKT pathway [56]. Although the *IRS1* mRNA abundance did not differ across the tissues studied, the *AKT1* mRNA abundance was higher in the small intestine, suggesting that the protein encoded by *AKT1* may not be regulated by IRS1. Compared with the duodenum and rumen, the fact that the *AKT1* and *MTOR* abundances were similar in the jejunum and ileum suggested that the mTOR pathway may be more active in these tissues through the regulation of AKT. The higher abundances of *AKT1* and *MTOR* mRNA in the small intestine than those in the rumen indicated a greater protein synthesis ability in the former. EEF1A1 delivers aminoacylated tRNAs to the ribosome, promoting elongation during translation [57,58]. Thus, despite the lower *AKT1* and *MTOR*, the greater *EEF1A1* mRNA abundance in the rumen compared with that in the small intestine in the present study suggested a robust elongation process during protein synthesis in this tissue [25].

Arg and Leu induce ribosomal protein S6 kinase beta-1 (S6K1) activation and the phosphorylation of EIF4EBP1 in sheep intestinal epithelial cells [43]. As we only measured the AA concentrations and mRNA abundance of *EIF4EBP1*, and not the abundance of phosphorylated EIF4EBP1, it is challenging to speculate on the relationship among those molecules. The supply of Gln can enhance mTOR signaling and protein synthesis in porcine intestinal epithelial cells [59,60]. Thus, the greater Gln, Arg, and Leu concentrations, and *MTOR* mRNA abundance in the small intestine than those in the rumen suggested the possibility that these three AAs are functionally correlated with MTOR protein activity.

### 4.3. mRNA Abundance of Amino Acid Transporters

The small intestine, a monolayer connected with tight junctions [61], is the primary location of AA absorption [62] and transports most nutrients from the lumen to the circulation. The AA transporters are located in both the apical membrane (on the lumen side) and basolateral membrane (mucosa side) to complete the transport of AA from the lumen to the blood [63]. In our study, although we did not measure the AA transporters in specific membranes, our data provided information on the mechanisms of AA transport and metabolism of dairy cows. Most of the mRNA abundances of AA transporters were greater in the small intestine than in the rumen, including *SLC1A1*, *SLC6A6*, *SLC7A8*, *SLC38A1*, *SLC38A7*, *SLC38A11*, and *SLC43A2*, indicating that these transporters are potentially more related to the absorption of AA and located in the apical membrane of intestinal epithelial cells. 

SLC1A1, also referred to as EAAT3 and EAAC1, is the major transporter of Glu and Asp [64,65]. Along with mTOR, its protein abundance increased in oocytes from the model organism Xenopus [66]. It is located in the plasma membrane in MDCKII cells and the apical membrane of human kidney tubules and proximal tubules [65]. *SLC1A1* was also detected in mammary tissue [67] and placentomes [6] from dairy cows. *SLC6A6* is a Na+- and Cl−-dependent, high-affinity, low-capacity transporter of taurine and β-alanine on the membrane surface [68]. The greater mRNA abundances of *SLC1A1* and *SLC6A6* in the small intestine than those in the rumen may be related to the fact that the Asp and taurine concentrations were greater, and the Glu concentration tended to be greater in the intestine compared with that in the rumen (as discussed in a previous section of this paper). SLC7A8, named LAT2, transports all of the L-isomers of neutral α-amino acids and it dimerizes with *SLC3A2* to achieve a functional expression in Xenopus oocytes. It exhibited higher affinity to Tyr, Phe, Trp, Thr, Asn, Ile, Cys, Ser, Leu, Val, and Gln, and relatively lower affinity to His, Ala, Met, and Gly [69]. In our study, its mRNA abundances in the jejunum and ileum were the highest, but another study in beef cattle revealed no significant differences among the duodenum, jejunum, and ileum [70]. SLC38A1, also named NAT2 and ATA1, is an important transporter of Gln [71] and, in humans, it is also specific for the transport of small short-chain, neutral AAs, such as Ala, Ser, Met, Asp, Gly, Pro, Thr, Leu, and Phe [72]. The knockdown of *SLC38A1* decreased the protein abundances of p70S6k1(T389), p-mTOR(S2448), and pS6(S235/236) in neurons from mice [73], which indicated that this AA transporter potentially affects protein synthesis through the mTOR pathway. In our study, the *SLC38A1* mRNA abundance was greater in the small intestine than in the rumen, which was similar to the profile of the *MTOR* mRNA abundance.

The protein *SLC38A7* on the cell membrane in the central nervous system transports not only L-Gln, but also L-His and L-Ala [74]. *SLC43A2* transports the branched-chain AAs Phe and Met, and is expressed in kidney tubule and small intestinal epithelial cells. The knockout of *SLC43A2* mice led to growth restriction, postnatal malnutrition, and early death [75]. We could not detect the mRNA abundance of solute carrier family 38 member 11 (*SLC38A11*) in the rumen, and its abundances were similar in the different sections of the GIT. Few data are available for the specific function of SLC38A11, and it is predicted to be an AA transporter. It belongs to the SLC38 family and, in non-ruminants, is expressed in the spleen, eye, marrow, and pharynx. The 11 members of the SLC38 family are Na+-dependent and carry out the net transport of neutral AAs [76]. Each of these AA transporters can handle several AAs, and in the present study, their mRNA abundance was greater in the small intestine than that in the rumen. Thus, along with the fact that most AA concentrations were greater in the small intestine than in the rumen, these data underscore the unique function of the small intestine in AA transport.

In our study, the mRNA abundance of *SLC7A5*, also referred to as *LAT1*, in the rumen was greater than that in the small intestine, but the *SLC7A5* mRNA abundance in the duodenum was greater than that in the rumen of dairy cows in another study [77]. In beef cattle, the mRNA abundance of *SLC7A5* in the duodenum was greater than that in the ileum [70]. *SLC7A5* transports the essential AAs and some hormones, such as dopamine and the thyroid hormones T3 and T4 [78]. SLC3A2, also known as CD98 or 4F2 heavy chain (4F2hc), dimerizes with several light chains of nutrient transporters, such as *SLC7A5* in the plasma membrane [69]. The *SLC3A2* mRNA abundances were not significantly different among the duodenum, jejunum, and ileum in a beef study and our study [70]. 

The AA transporters in cells can have a direct impact on the protein synthesis pathways; for instance, the knockdown or knockout of solute carrier family 3 member 2 (SLC3A2/CD98hc) and solute carrier family 7 member 5 (SLC7A5), controlling essential AA transport, inhibited the mTOR pathway [79,80]. In humans, the *SLC1A5* gene, known as ASCT2, encodes a sodium-dependent neutral AA antiporter, which mainly exchanges Gln with other neutral AAs, such as Ser, Asn, or Thr [2]. The transport of Ala and Gly was greater in a human placental choriocarcinoma cell line transfected with *SLC1A5* cDNA [81]. A previous study revealed that its mRNA abundance was greater in the duodenum than in the rumen [73], while the *SLC1A5* mRNA abundance was greater in the small intestine. Thus, the fact that the mRNA abundances of *SLC7A5, SLC3A2,* and *SLC1A5* were greater in the rumen in the present study suggested that these two AA transporters may be biologically important in this tissue. 

Except for *SLC1A1*, *SLC38A1*, *SCL3A1*, and *SLC7A5* mentioned above, lysosomal AA transporters, such as *SLC7A11* [82], *SLC38A9* [83,84,85], *SLC15A4* [86], *SLC36A4*, and *SCL36A1* [87,88], also regulate the mTOR pathway or protein synthesis. The mRNA abundances of *SLC1A1* and *SLC1A5* in the fetal and adult lamb intestine were altered by feed restriction, feeding rumen-protected Arg, and also feeding N-carbamylglutamate [8,89]. The abundances of *SLC1A1*, *SLC1A5*, *SLC3A2*, *SLC7A5*, and *SLC38A1* mRNA were detected in the adipose tissue [4,5] of dairy cows during the transition into lactation. In fact, feeding RPM enhanced the mRNA abundances of *SLC1A1*, *SLC1A5*, *SLC3A2*, *SLC36A1*, and *SLC38A1*. In the same study, the mRNA abundance of *SLC1A1* was altered at different time points around parturition. The SLC1A3 protein abundance was also higher in the group fed RPM, while the protein abundances of *SLC38A1* and *SLC1A5* did not differ between the controls and the cows fed RPM. The protein abundance of these three targets changed to different extents around parturition [4]. In an in vitro study, the mRNA abundances of *SLC7A5* and *SLC3A2* were decreased by Arg supplementation, and a challenge with lipopolysaccharide also decreased the abundance of *SLC3A2* [7]. In the placenta of dairy cows, the mRNA abundances of *SLC3A2*, *SLC7A5*, *SLC38A1*, and *SLC43A2* were altered by feeding RPM [6]. Similarly, the *SLC1A1*, *SLC1A5*, *SLC3A2*, *SLC7A5*, and *SLC38A2* mRNA abundances were affected by Arg and Met supplementation in an in vitro study with bovine mammary epithelial cells [20]. Together, these data strongly suggested that the mRNA abundance of AA transporters is affected by nutritional factors. Thus, in addition to the absorptive function carried out by each section of the GIT, it is likely that the dietary supply of AA interacts with the GIT in order to coordinate AA use.

## 5. Summary and Conclusions

Except for Asn, Cys, and Gly, the AA concentrations were greater in the small intestine compared with those in the rumen, which seems to agree with the purported absorptive function of each section of the GIT. It is noteworthy, however, that the present data underscored how components of the epithelium, e.g., keratinized ruminal cells, along with pathways to generate urea and creatine, are potential factors that shape the tissue profiles of AA. The greater *MTOR* and *AKT1* mRNA abundances in the small intestine suggested that it has a greater protein synthesis ability, while the greater *EEF1A1* mRNA abundance in the rumen suggested the existence of a robust elongation process. Most of the AA transporters measured, including *SLC1A1*, *SLC6A6*, *SLC7A8*, *SLC38A1*, *SLC38A7*, *SLC38A11*, and *SLC43A2*, had greater abundances in the small intestine than those in the rumen. This underscored their essential role in allowing for AA transport in the small intestine. Along the same lines, the greater mRNA abundances of *SLC7A5, SLC3A2,* and *SLC1A5* suggested that they play unique roles in ruminal tissue function. Overall, the present study uncovered novel relationships between tissue-specific AA profiles and the abundance of AA transporters, and served as the basis for further research. Potential areas of study include assessing the role of nutrition in absorptive mechanisms across the GIT, not only to determine the utilization of AA within the tissue, but also the degree to which they enter the circulation and provide substrates for peripheral tissues. 

## Figures and Tables

**Table 1:** Least-squares means, pooled SEM, and *p*-values for amino acid concentrations in tissue harvested from the rumen, duodenum, jejunum, and ileum from lactating Holstein cows (*n* = 8/group).

Item, ng/mg of Total Protein	Rumen	Duodenum	Jejunum	Ileum	SEM	*p*-Value	Contrast *p* Value		
							Rumen vs. Small Int	Duodenum vs. Jejunum and Ileum	Jejunum vs. Ileum
Alanine	8652 ^b^	13,297 ^a^	13,724 ^a^	12,508 ^a^	1123	<0.01	<0.01	0.87	0.35
Arginine	136 ^b^	2975 ^a^	2824 ^a^	2298 ^a^	248	<0.01	<0.01	0.13	0.10
Asparagine	600 ^a^	332 ^b^	314 ^b^	207 ^b^	34	<0.01	<0.01	0.09	0.03
Aspartate	2111 ^c^	11,228 ^a^	7158 ^b^	7362 ^b^	1093	<0.01	<0.01	<0.01	0.88
Cysteine	5173 ^a^	1757 ^b^	2695 ^ab^	2426 ^b^	658	<0.01	<0.01	0.31	0.77
Cystine	701	37	457	317	199	0.13	0.06	0.15	0.60
Glutamine	3450 ^b^	6182 ^a^	7154 ^a^	5695 ^a^	692	<0.01	<0.01	0.69	0.05
Glutamate	21,486	23,149	26,447	20,688	2325	0.09	0.31	0.84	0.02
Glycine	10,804 ^ab^	13,801 ^a^	10,053 ^bc^	7155 ^c^	899	<0.01	0.64	<0.01	0.03
Histidine	447 ^b^	1099 ^a^	1016 ^a^	731 ^b^	85	<0.01	<0.01	0.02	0.01
Isoleucine	1981 ^b^	3841 ^a^	3722 ^a^	2856 ^ab^	298	<0.01	<0.01	0.11	0.03
Leucine	2938 ^b^	7424 ^a^	7124 ^a^	5559 ^a^	573	<0.01	<0.01	0.09	0.04
Lysine	5573 ^b^	7738 ^a^	8090 ^a^	6165 ^ab^	740	0.01	0.01	0.34	0.01
Methionine	734 ^c^	4349 ^a^	3392 ^ab^	2615 ^b^	386	<0.01	<0.01	<0.01	0.09
Phenylalanine	1447 ^c^	5866 ^a^	5251 ^ab^	4140 ^b^	474	<0.01	<0.01	0.03	0.07
Proline	3271 ^b^	5165 ^a^	5985 ^a^	4591 ^ab^	513	<0.01	<0.01	0.79	0.02
Serine	546 ^b^	3400 ^a^	3442 ^a^	2937 ^a^	280	<0.01	<0.01	0.45	0.13
Taurine	4540 ^c^	15,224 ^b^	20,913 ^a^	20,826 ^a^	1610	<0.01	<0.01	<0.01	0.96
Threonine	1520 ^b^	3443 ^a^	3854 ^a^	3226 ^a^	329	<0.01	<0.01	0.76	0.10
Tryptophan	652 ^b^	993 ^a^	764 ^ab^	507 ^b^	96	<0.01	0.28	<0.01	0.04
Tyrosine	1744 ^b^	6414 ^a^	6170 ^a^	5006 ^a^	551	<0.01	<0.01	0.15	0.08
Valine	4953 ^b^	7724 ^a^	8305 ^a^	6618 ^ab^	599	<0.01	<0.01	0.66	0.02

^a,b,c^ Means on the same row differ (*p* < 0.05).

**Table 2:** Least-squares means, pooled SEM, and *p*-values for the mRNA abundances of target genes associated with protein synthesis and amino acid transport in tissue harvested from the rumen, duodenum, jejunum, and ileum from lactating Holstein cows (*n* = 8/group).

Item ^1^	Rumen	Duodenum	Jejunum	Ileum	SEM	*p*-Value	Contrast *p* Value		
							Rumen vs. Small Int	Duodenum vs. Jejunum and Ileum	Jejunum vs. Ileum
Protein synthesis									
*IRS1*	1.23	1.09	0.87	1.66	0.22	0.11	0.93	0.54	0.02
*AKT1*	0.74 ^b^	1.14 ^b^	1.66 ^a^	1.82 ^a^	0.11	<0.01	<0.01	<0.01	0.31
*MTOR*	0.61 ^c^	1.05 ^b^	1.6 ^a^	1.77 ^a^	0.10	<0.01	<0.01	<0.01	0.09
*EIF4EBP1*	0.85	0.96	0.62	0.89	0.11	0.16	0.86	0.14	0.09
*EIF4EBP2*	1.11	1.03	0.93	1.18	0.07	0.12	0.48	0.38	0.02
*RPS6KB1*	1.07	0.96	1.24	0.91	0.13	0.30	0.82	0.48	0.08
*EEF1A1*	1.51 ^a^	0.93 ^b^	0.80 ^b^	0.99 ^b^	0.06	<0.01	<0.01	0.65	0.04
Amino acids transporters									
*SLC1A1*	0.05 ^b^	0.44 ^ab^	4.1 ^a^	3.71 ^ab^	1.00	0.01	0.02	0.01	0.77
*SLC1A5*	1.26	1.00	0.69	1.05	0.14	0.06	0.04	0.47	0.08
*SLC3A2*	1.51 ^a^	0.83 ^b^	0.97 ^b^	1.11 ^b^	0.08	<0.01	<0.01	0.03	0.2
*SLC6A6*	0.01 ^c^	0.24 ^bc^	3.4 ^a^	3.14 ^ab^	0.84	0.01	0.02	<0.01	0.81
*SLC7A5*	1.28 ^a^	1.25 ^b^	0.52 ^b^	0.71 ^ab^	0.16	<0.01	0.02	<0.01	0.41
*SLC7A8*	0.01 ^c^	0.28 ^bc^	3.88 ^a^	3.33 ^ab^	0.82	<0.01	0.02	<0.01	0.63
*SLC38A1*	0.34 ^b^	1.28 ^a^	0.86 ^a^	1.28 ^a^	0.13	<0.01	<0.01	0.19	0.03
*SLC38A2*	1.12	1.07	0.86	1.28	0.09	0.66	0.60	0.70	0.28
*SLC38A7*	0.76 ^b^	1.41 ^a^	1.56 ^a^	1.69 ^a^	0.10	<0.01	<0.01	0.08	0.35
*SLC38A11*	NA	2.52	1.56	1.69	0.19	0.98	NA	0.89	0.89
*SLC43A2*	0.08 ^b^	0.73 ^ab^	3.83 ^a^	4.02 ^a^	0.91	0.01	0.01	0.01	0.88

^a,b,c^ Means in the same row differ (*p* < 0.05). ^1^ Full description of gene symbols. IRS1, insulin receptor substrate 1; AKT1Akt1, AKT serine/threonine kinase 1; mTOR, mechanistic target of rapamycin kinase; RPS6KB1, ribosomal protein S6 kinase B1; EIF4BP1, eukaryotic translation initiation factor 4E-binding protein 1; EIF4EBP2, eukaryotic translation initiation factor 4E binding protein 2; EEF1A1, eukaryotic translation elongation factor 1 alpha 1; SLC1A1, solute carrier family 1 member 1; SLC1A5, solute carrier family 1 member 5; SLC3A2, solute carrier family 3 member 2; SLC6A6, solute carrier family 6 member 6; SLC7A5, solute carrier family 7 member 5; SLC7A8, solute carrier family 7 member 8; SLC38A1, solute carrier family 38 member 1; SLC38A2, solute carrier family 38 member 2; SLC38A7, solute carrier family 38 member 7; SLC38A11, solute carrier family 38 member 11; SLC43A2, solute carrier family 43 member 2.

## Data Availability

Data presented in this study are available upon reasonable request from the corresponding author (J.J.L).

## Supplementary Materials

The following supporting information can be downloaded at: https://www.mdpi.com/article/10.3390/ani13071189/s1, Supplementary File: Supplemental Methods; Table S1: GenBank accession number, sequence, and amplicon size of primers for Bos taurus used to analyze gene expression; Table S2: Median Ct, Median ∆Ct Slope, coefficient of determination of the standard curve (R^2^), efficiency of amplification, relative mRNA abundance, 1/E∆Ct, and percentage of mRNA abundance; Figure S1: Boxplot of taurocholic acid, glycocholic acid, and glycochenodeoxycholic acid concentrations in tissue harvested from the rumen, duodenum, jejunum, and ileum of lactating Holstein cows (*n* = 8/group).
