# Supplementary material for: Abundance of Amino Acid Transporters and mTOR Pathway Components in the Gastrointestinal Tract of Lactating Holstein Cows
Source: Animals (Basel). 2023 Mar 29;13(7):1189. doi: 10.3390/ani13071189 (PMC10093496; doi:10.3390/ani13071189)

Supplemental Methods

RNA extraction
Approximately 50 mg of tissue was homogenized with 1 mL Qiazol (Qiagen, Hilden, Germany). Samples were centrifuged for 10 min at 12,000 × g at 4°C, and the supernatant was collected and held at room temperature for 5 min. Two-hundred µL chloroform was added, shaken by hand, incubated at room temperature for 3 min, and centrifuged for 15 min at 12,000 × g at 4°C. The supernatant was collected and mixed with 750 µL ethanol. The samples were centrifuged at 12,000 rpm for 15 sec in the miRNeasy mini spin column to collect the pellet. Three-hundred fifty µL RWT buffer was used to wash the sample, followed by centrifugation at 12,000 rpm for 15 s. Eighty µL DNase I digestion mix (DNase I: RDD buffer = 1:7) (Qiagen, Hilden, Germany) was added to the spin column to degrade DNA. Three-hundred fifty µL RWT buffer was used to wash the sample, followed by centrifugation at 12,000 rpm for 15 s. Five-hundred µL RPE buffer was used to wash the sample twice for 15 seconds, followed by centrifugation at 12,000 rpm for 2 min. The miRNeasy mini spin column was placed in a new 2 mL collection tube, and centrifuged at 15,000 rpm for 1 min. The miRNeasy mini spin column was transferred to a new 1.5 mL collection tube, and 50 µL of RNase-free water was added directly on to mini spin column membrane. After incubating for 1 min, the column was centrifuged for 1 min at 12,000 rpm to elute RNA. Total RNA quantification was determined using a Nanodrop ND-1000 (NanoDrop Technologies, Rockland, DE). The RNA was diluted to 100 ng/µL with DNase/RNase-free water. The purity and integrity of extracted RNA were evaluated using an Agilent Bioanalyzer at the ROY J. CARVER Biotechnology Center, University of Illinois, Urbana-Champaign.

qPCR
qPCR was performed in a MicroAmp Optical 384-Well Reaction Plate (Cat. #4309849, Applied Biosystems). Within each well, 4 µL of diluted cDNA combined with 6 µL of a mixture composed of 5 µL 1×SYBR Green master mix (Cat. #4309155, Applied Biosystems), 0.4 µL each of 10 µM forward and reverse primers, and 0.2 µL of DNase/RNase-free water were added. To test the relative expression level, three replicates and a 7-point standard curve plus the nontemplate control (NTC) were run for each sample. qPCR was conducted in QuantStudio 7 real-time PCR machine (Applied Biosystems) following the conditions: 2 min at 50°C, 10 min at 95°C, 40 cycles of 15 s at 95°C (denaturation), and 1 min at 60°C (annealing + extension). The presence of a single PCR product was verified by the dissociation protocol using incremental temperatures to 95°C for 15 s, then 60 °C for 1min. The threshold cycle (Ct) and quantity data were analyzed and transformed using the standard curve with the QuantStudio Software (version v1.7.2, Applied Biosystems, CA). Quantity data were normalized by dividing the geometric mean of the three Internal Control Genes (ICG): UXT, GAPDH, and RPS9. Each gene was run in triplicate.

Relative mRNA Abundance
The Ct value, slope, and R^2 data were analyzed by QuantStudio Software (version v1.7.2, Applied Biosystems, CA). The efficiency of qPCR amplification for each gene was calculated using the standard curve method (Efficiency = $10^{(-1/slope)}$). Relative mRNA abundance among measured genes was calculated by: 1/(Efficiency of tested gene × Median of ΔCt of tested gene in all samples). The ΔCt of tested genes for each sample was calculated by: Ct value of tested

gene in each tested sample – geometric mean Ct of 3 ICG in each tested sample. The overall percentage of relative mRNA abundance (mRNA abundance%) was computed from the equation: 100 × mRNA abundance of each gene/sum of mRNA abundance of all the genes investigated.

Supplemental table

Supplemental Table 1. GenBank accession number, sequence, and amplicon size of primers for Bos taurus used to analyze gene expression.

Gene	Accession #	Primers (5'-3')	bp
Amino acid transport			
SLC1A1	NM_174599.2	GGTGATTGTGCTGAGTGCTG<br>CTCCACAATGCCAGTCCCAA	133
SLC1A5	NM_174601.2	GGCTAGCAGCTGTTTACTCCT<br>AGTCTGGGGGCTAGAAGACG	129
SLC3A2	NM_001024488.2	GAGCATTCCCTTGCTTGCAC<br>GCTCATGGTGCCTGAGTCG	175
SLC6A6	NM_174610.2	TCGCCAACCTGACGGTGTTA<br>GGCTTCAGGATGTCCTTGTGG	118
SLC7A5	NM_174613.2	CCGTACCCTCACTGGTGTTC<br>AGATGAACCTTGATGGGCCG	181
SLC7A8	NM_001192889.2	GCACCGGAACAACACTGAAA<br>CAGAGCCGATGATGTTCCCTAC	143
SLC38A1	XM_010827702.2	GGAAGGGCGGATACCACTTT<br>TGACACCCCTGTTATCTCAGC	168
SLC38A2	NM_001082424.1	TGAAAAGCCATTATGCCGATGT<br>CCCACAATCGCATTGCTCAG	148
SLC38A7	NM_001100355.1	CTTTGTCTTCCCAGGGCTGT<br>CCCAGTGTGACCAAGAGGAC	114
SLC38A11	NM_001031760.1	TAGAGCTCAATGGCGTGCTC<br>AGCACCAATGGGAAGCATGA	134
SLC43A2	NM_001075546.1	TACACCTCCATCTTCGGGGT<br>TTCTTCTCACCTGGGTTGGC	143
mTOR pathway			
AKT1	NM_173986.2	CTGCACAAGCGAGGTGAGTA<br>GAGAAGTTGTTGAGGGGCGA	134
MTOR	NM_001144096.3	GGTTAACACCAAGCAGGTTCAT<br>GTATGTCGCACTGGACACCA	134
RPS6KB1	NM_205816.1	ACAGCCTGCTTTTACTTGGC<br>AGGTGTGTGTGACTGTTCCG	178
EIF4EBP1	NM_001077893.2	GGAGTGTCGGAACTCACCTG<br>AACTGTGACTCTTCACCGCC	162
EIF4EBP2	NM_001191149.1	AAGCATGCAGTTGGGGATGA<br>AACGGGGATTGCTGGTACTG	158
EEF1A1	NM_174535.2	CCCTTCCTGGGGACAATGTG	134

		AATAATCACCTGAGCTGTGAAGC	
IRS1	XM_003581871.4	CTCAAGAGTGCCCACCTCAA<br>AGGTCTTCATTCTGCTGTGAT	187

Supplemental Table 2. Median Ct, Median ΔCt Slope, coefficient of determination of the standard curve (R2), efficiency of amplification, relative mRNA abundance, 1/EΔCt and percentage of mRNA abundance.

Gene	Median Ct[1]	Median ΔCt[2]	Slope[3]	(R^2)[4]	Efficiency[5]	relative mRNA abundance[6]	mRNA abundance%[7]
mTOR	24.88	4.13	-3.44	0.99	1.95	0.06	0.41
Akt	22.92	2.28	-3.27	1.00	2.02	0.20	1.31
RPS6KB1	25.09	4.46	-3.59	0.96	1.90	0.06	0.37
EIF4BP1	25.24	4.76	-3.51	0.96	1.93	0.04	0.29
EIF4EBP2	21.93	1.28	-3.38	0.98	1.97	0.42	2.71
EEF1A1	16.84	-3.93	-3.46	0.99	1.95	13.73	88.93
IRS1	26.39	5.64	-3.04	0.98	2.13	0.01	0.09
SLC1A1	26.07	6.13	-3.37	0.98	1.98	0.02	0.10
SLC1A5	24.10	3.49	-3.65	0.98	1.88	0.11	0.72
SLC3A2	26.44	5.62	-3.48	0.98	1.94	0.02	0.16
SLC6A6	28.41	7.72	-3.66	0.98	1.88	0.01	0.05
SLC7A5	24.98	4.26	-3.43	0.96	1.96	0.06	0.37
SLC7A8	27.15	6.53	-3.37	0.97	1.98	0.01	0.07
SLC38A1	24.49	3.73	-3.30	0.99	2.01	0.07	0.48
SLC38A2	21.45	0.98	-3.37	1.00	1.98	0.51	3.32
SLC38A7	26.25	5.52	-3.63	0.94	1.88	0.03	0.20
SLC38A11	27.93	6.59	-3.48	0.99	1.94	0.01	0.08
SLC43A2	24.90	4.23	-3.34	0.99	1.99	0.05	0.35

[1] The median was calculated considering all samples.

[2] The median of ΔCt was calculated as [Ct gene – geometrical mean of Ct internal controls] for all samples.

[3] Slope of the standard curve.

[4] R2 stands for the coefficient of determination of the standard curve.

[5] Efficiency was calculated as $[10^{(-1 / \text{Slope})}]$.

[6] relative mRNA abundance = 1/ Efficiency Median ΔCt

[7] $1/E^{\Delta Ct}$ = 100 × (relative mRNA abundance/ ∑ relative mRNA abundance)

Supplemental Figure 1. Boxplot of taurocholic acid, glycocholic acid, and glycochenodeoxycholic acid concentrations in tissue harvested from rumen, duodenum, jejunum, and ileum from lactating Holstein cows (n = 8/group).

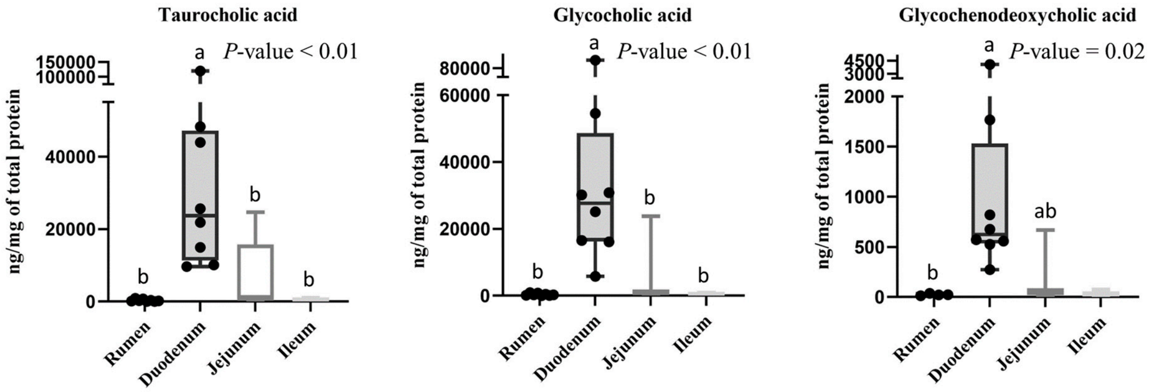

Supplement: Supplementary file 1 [file animals-13-01189-s001.zip › animals-2249947-supplementary.pdf]
